# The Anæsthetic Revelation

**Published:** 1889-04

**Authors:** J. H. Seymour


### THE ANÆSTHETIC REVELATION.

About two hours ago I was at New Haven undergoing an operation for the relief of my shoulder. The circumstances of the case were these:

Since December last, when my shoulder was dislocated by a fall from a tree, the motions of my left arm have been cramped and confined. Upon examination, the doctor said that in the healing of the large rent made in the ligaments of the joint, adhesions had formed between the ligaments and the bone so as to interfere with the free motion of the bone in its socket. He told me that I could secure freedom of the joint by persevering in a long process of gymnastics, or by taking ether and having the ligaments broken up; or, if I chose, I might lie right down on the lounge, and let him perform the operation at once without the ether.

It took but little reflection to enable me to choose the ether process. Soon after I commenced breathing the ether, as I lay on the lounge, I began to experience sensations utterly new and difficult to describe, though I will attempt it. I felt that the life was quitting my extremities all around, and concentrating at the center of my body. Imagine an oyster with his shell open, just withdrawing his inner self within its covering and gradually closing it, leaving nothing but inert matter outside. A moment came when the last conscious wag of the tongue was possible, and with it I said "Good-bye." I was fully conscious that I was about to launch into an unknown sea. The complete log-book of that cruise is certainly not in my memory, but I find some faint traces of it which I will endeavor to give.

I seemed to be in the presence of a multitude of persons who were being arranged by some mystical agency in a series of classes or ranks, like soldiers drilling. In some inexplicable way this drill work was identified with the great work of God, and his purpose in creating the universe. I was a passive subject of certain evolutions in which I was placed for a short time in one class or rank, and then for a short time in another. Mr. N. had much of the care of marshaling us here and there, and Mr. B., the noted almanac maker, stood by with pencil and paper, full of intense calculations and mathematical estimates of the results of each of these evolutions. The stake that was involved in these movements was nothing short of the everlasting settlement of the destiny of the human race, and the casting out of evil. The spirit that pervaded the whole movement was that of intense care and precision in making up and drilling the different classes, and enthusiastic bursts of rapture, applause and laughter followed each successive arrangement as it was presented to view.

Regularly and rapidly a certain crisis approached. Just at this stage of the proceedings two lines of verses were put into my mind, one of which terminated with "Ha, ha, ha," and the other with "Hurrah, hurrah," and I thought that I was appointed to finish the whole performance by repeating this doggerel.

I wondered much at this arrangement, but said I would obey. I spoke the first word or two of it. Instantly the whole drama of the universe broke in upon my spiritual vision like a lightning flash. I was conscious that when I should finish the last hurrah, the work of the world and the universe, so far as it had anything to do with evil, would be finished, and my cheers would be joined by those of untold millions, surging up from the hearts of the redeemed of the human race. You may be sure that I gave that last hurrah with an emphasis. Those in the room can testify to that. But it was scarcely out of my mouth before consciousness returned.

I was afterwards told that my trance lasted about twenty minutes: that about five minutes after I had said "good-bye," the doctor gradually, and by repeated efforts, forced my arm up over my head and turned the bone in its socket in every direction and that it was accompanied by an expression as of intense pain. When this was done with I began to laugh, and the laughter was accompanied with unintelligible talk relating to my dreams. I was afterwards impressed with a deep, solemn feeling, as if I had been intrusted with some of the deepest secrets of the universe. The after effects of the ether on my body seemed pleasant and stimulating.—J. H. SEYMOUR, in *Popular Science News*.

